# Investigation of the Anti-Lung Cancer Mechanisms of *Taraxacum officinale* Based on Network Pharmacology and Multidimensional Experimental Validation

**DOI:** 10.3390/ph18050663

**Published:** 2025-04-30

**Authors:** Jianing Liu, Hailing Yang, Ran Liu, Dongjin Sun, Yongbao Liu, Jing Lu, Jinbiao Liu, Junrui Lu

**Affiliations:** 1 School of Chemical Engineering and Technology, Tianjin University of Technology, Tianjin 300384, China; jianingliu1023@163.com (J.L.); 13865771672@163.com (H.Y.); cynthial9@163.com (R.L.); sundongjin@stud.tjut.edu.cn (D.S.); yj20230948@stud.tjut.edu.cn (Y.L.); 2 Analysis and Testing Center, Tianjin University of Technology, Tianjin 300384, China; lujing@email.tjut.edu.cn

**Keywords:** *Taraxacum officinale*, taraxasterol, lung cancer, network pharmacology, molecular docking, molecular dynamics simulation, in vitro cell assay, scanning electron microscopy

## Abstract

**Background:***Taraxacum officinale*(commonly known as dandelion) is a medicinal and edible plant, with the entire plant being used for therapeutic purposes. Studies have demonstrated that dandelion exhibits inhibitory effects against various types of cancer. However, research on its potential for lung cancer (LC) treatment is limited, and the specific compounds responsible for its anticancer effects, as well as the underlying mechanisms, remain unclear. **Methods**: This study aimed to elucidate the underlying pharmacological mechanisms by which dandelion exerts therapeutic effects against LC. Initially, active compounds of dandelion and their corresponding targets were retrieved from public databases. Subsequently, network pharmacology approaches were applied to identify LC-associated disease targets. By integrating drug-specific targets and disease-related targets, a comprehensive dandelion–lung cancer interaction network was established. Protein–protein interaction (PPI) analyses and functional enrichment studies were further performed to uncover potential molecular mechanisms. Additionally, molecular docking and molecular dynamics (MD) simulations were conducted to evaluate binding interactions between critical active constituents and core targets. To experimentally validate these findings, in vitro cellular assays combined with scanning electron microscopy (SEM) were employed to investigate the anticancer effects of taraxasterol, a key bioactive sterol compound isolated from dandelion, on LC cells. **Results**: Our analyses identified 58 active compounds in dandelion linked to 614 potential targets, of which 228 targets were associated with LC. The PPI network highlighted 16 core targets, notably TP53, CASP3 and EGFR. Functional enrichment analysis suggested that dandelion might exert its anticancer effects by modulating the tumor microenvironment through the regulation of these critical targets. Molecular docking results demonstrated stable binding interactions between major active compounds and the identified core targets. Furthermore, the anticancer activity of taraxasterol was experimentally validated through in vitro assays and SEM-based morphological assessments, confirming its inhibitory effects on A549 lung cancer cells. **Conclusions**: Collectively, our findings reveal a multi-targeted therapeutic mechanism of dandelion against LC and support its potential development as a novel natural candidate for lung cancer treatment.

## 1. Introduction

According to global cancer statistics, lung cancer ranks first in both incidence and mortality among all malignant tumors, posing a significant threat to global public health. Data from GLOBOCAN 2020 indicate that LC accounts for approximately 12.4% of all newly diagnosed cancers and contributes to 18% of total cancer-related deaths worldwide [1]. Although various treatment strategies—such as surgery, chemotherapy, radiotherapy, targeted therapy, and immunotherapy—are widely applied in clinical settings [2], their efficacy remains limited due to issues such as drug resistance, high recurrence rates, severe side effects, and substantial treatment costs. In particular, the survival rate of patients with advanced LC remains poor, highlighting the urgent need for novel therapeutic strategies that are both effective and low in toxicity, while maintaining cost-efficiency.

In recent years, natural products have gained increasing attention in cancer therapy owing to their multi-target and multi-mechanism characteristics [3,4]. Dandelion, a traditional edible and medicinal plant, has demonstrated anti-inflammatory, antioxidant, and antitumor properties, and exhibits potential therapeutic value in the treatment of various malignancies [5,6]. Previous studies have shown that extracts of dandelion exert anticancer effects through multiple mechanisms, including induction of apoptosis [7,8], inhibition of cell proliferation, regulation of oxidative stress, and modulation of the tumor microenvironment [9,10]. However, the specific molecular mechanisms underlying its therapeutic potential in LC remain inadequately studied, and further systematic investigation is warranted to elucidate its key targets and pharmacological pathways.

Network pharmacology [11,12], as a powerful tool for elucidating the complex mechanisms of traditional herbal medicines, enables the systematic identification of potential targets and signaling pathways involved in multi-component therapeutic actions. In addition, molecular docking [13,14] and molecular dynamics (MD) simulations [15,16] can provide theoretical support for drug–target interactions by evaluating the binding affinity and stability between active compounds and core targets.

In this study, we utilized network pharmacology approaches to screen for active components of dandelion with potential anti-LC activity and identify their corresponding targets. Gene Ontology (GO) enrichment, Kyoto Encyclopedia of Genes and Genomes (KEGG) pathway analysis, and protein–protein interaction (PPI) network construction were subsequently conducted to explore the underlying molecular mechanisms. Molecular docking and MD simulations were then performed to assess the binding interactions between major active components and key targets. Finally, in vitro cell-based assays [17] and scanning electron microscopy (SEM) analysis [18] were used to validate the anti-LC activity of taraxasterol, a representative sterol compound derived from dandelion. This study aims to provide a scientific basis for the application of dandelion in LC treatment and offer new insights into the multi-target mechanisms of natural products in cancer therapy.

## 2. Results

### 2.1. Network Pharmacology Research

#### 2.1.1. Key Active Compounds of Dandelion

**Figure 1:**
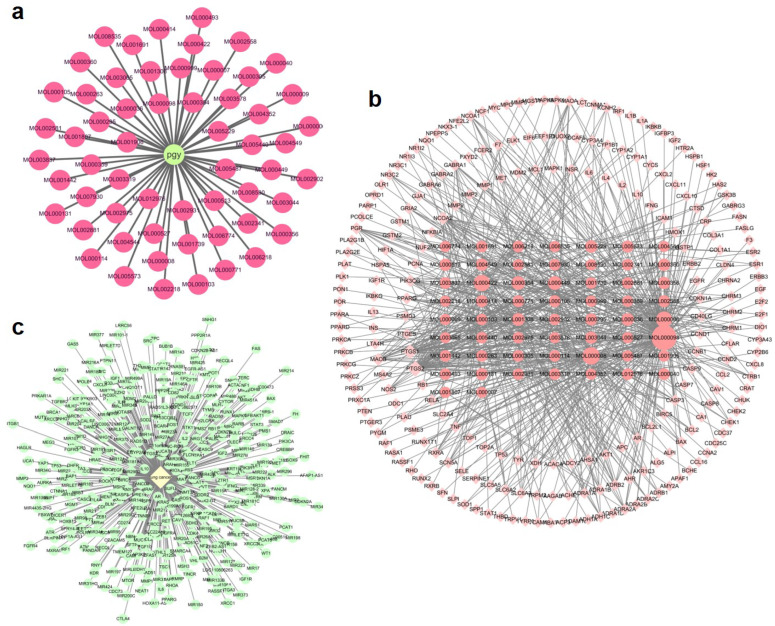
Network pharmacology analysis. (**a**) Herb–compound network. (**b**) Compound–target interaction network. (**c**) LC-target network.

**Figure 2:**
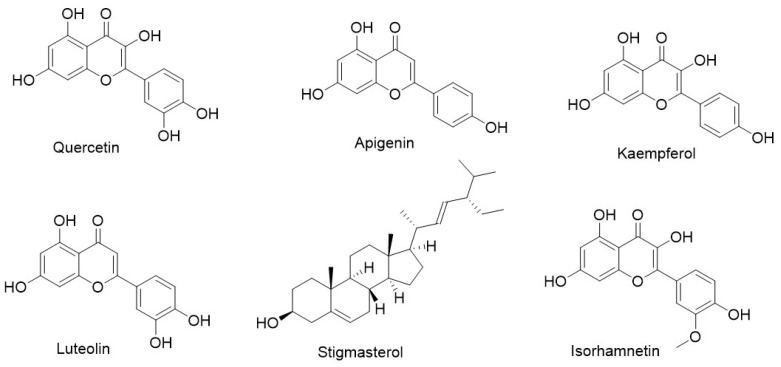
Chemical structures of key compounds.

Active compounds of dandelion were screened from the Traditional Chinese Medicine Systems Pharmacology (TCMSP) database using the criteria of oral bioavailability (OB) ≥ 30% and drug-likeness (DL) ≥ 0.18. In addition, compounds that have been explicitly reported in the literature to exhibit anticancer activity or possess anticancer potential were also included. A total of 58 active compounds and 228 lung cancer-related targets were identified, yielding 664 compound–target interaction pairs. Cytoscape 3.8.2 software was used to construct a herb–compound network (Figure 1a) and a compound–target network (Figure 1b). In the compound–target network (Figure 1b), nodes are sized according to their degree values, with larger nodes representing higher degrees and greater centrality within the network. Based on degree ranking, the top six key compounds were identified (Figure 2), namely MOL000098 (quercetin, degree = 132), MOL000008 (apigenin, degree = 61), MOL000422 (kaempferol, degree = 51), MOL000006 (luteolin, degree = 48), MOL000449 (beta-sitosterol, degree = 27), and MOL000354 (isorhamnetin, degree = 25).

#### 2.1.2. Key Active Compounds and Their Targets Associated with LC

LC-associated targets were systematically retrieved from GeneCards, UniProt, and DisGeNET databases. Following filtration based on a relevance score ≥ 40, 416 potential disease-related targets were identified (Figure 1c).

#### 2.1.3. Intersection Targets Between Dandelion and LC

**Figure 3:**
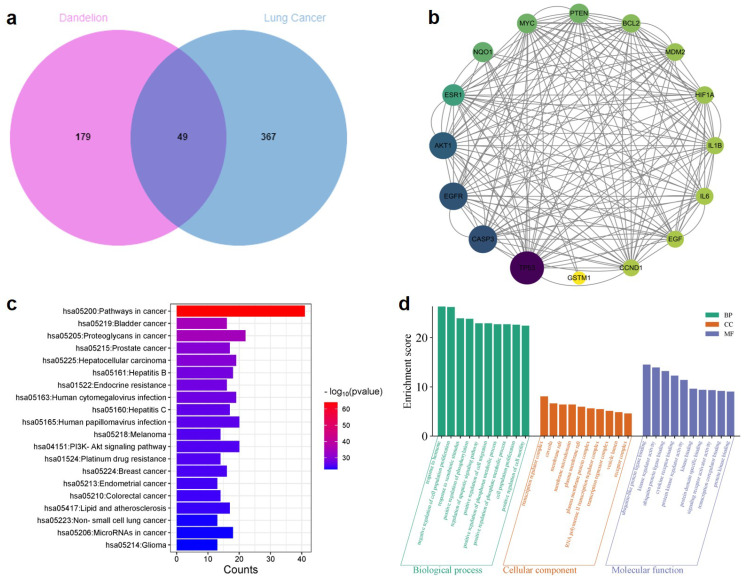
Network-based functional enrichment and interaction analysis. (**a**) Overlapping targets between dandelion and LC. (**b**) PPI network of the overlapping targets. (**c**) GO enrichment analysis of overlapping targets. (**d**) KEGG pathway enrichment analysis of overlapping targets.

A Venn diagram was constructed using the Bioinformatics online platform (https://www.bioinformatics.com.cn, accessed on 6 August 2024). This diagram illustrates the overlapping targets between the predicted anticancer targets of active components from dandelion (228 targets) and LC-related targets (416 targets). As shown in Figure 3a, a total of 49 common targets were identified.

#### 2.1.4. PPI Network Analysis of Core Targets Associated with the Anti-LC Effects of Dandelion

The 49 overlapping targets were imported into the STRING database for protein–protein interaction (PPI) analysis. The resulting PPI network was visualized using Cytoscape 3.8.2 software (Figure 3b). Subsequently, key nodes were screened using CytoNCA analysis with a betweenness centrality (BC) threshold ≥ 40. This analysis yielded a refined network comprising 16 nodes and 176 edges. In the visualized network, nodes with higher BC values are depicted as larger and darker, reflecting their greater centrality, and functional significance.

#### 2.1.5. GO and KEGG Analysis

The overlapping genes were imported into the Metascape database for enrichment analysis. Terms with a screening criterion of *p* < 0.05 were considered statistically significant, and the top 10 enriched terms from each GO category—including molecular function (MF), biological process (BP), and cellular component (CC)—were selected based on ascending *p*-values. The results of GO enrichment are presented as bar graphs (Figure 3c). The GO enrichment results revealed that, at the molecular function level, the anti-LC mechanisms of dandelion mainly involve ubiquitin-like protein ligase binding, kinase regulator activity, ubiquitin protein ligase binding, and cytokine receptor binding. At the biological process level, the enriched terms primarily include response to hormone, negative regulation of cell population proliferation, response to external stimulus, positive regulation of phosphorylation, regulation of apoptotic signaling pathways, and positive regulation of cell migration. Regarding cellular components, the key targets were mainly associated with transcription regulator complexes, caveolae, membrane rafts, and membrane microdomains. In addition, the top 20 pathways identified through KEGG pathway analysis were visualized as bar graphs (Figure 3d). The results indicate that the potential overlapping targets are closely related to several cancer-associated pathways, including pathways in cancer, bladder cancer, proteoglycans in cancer, prostate cancer, PI3K-Akt signaling pathway, non-small-cell-lung cancer, and glioma.

### 2.2. Molecular Docking Results

**Figure 4:**
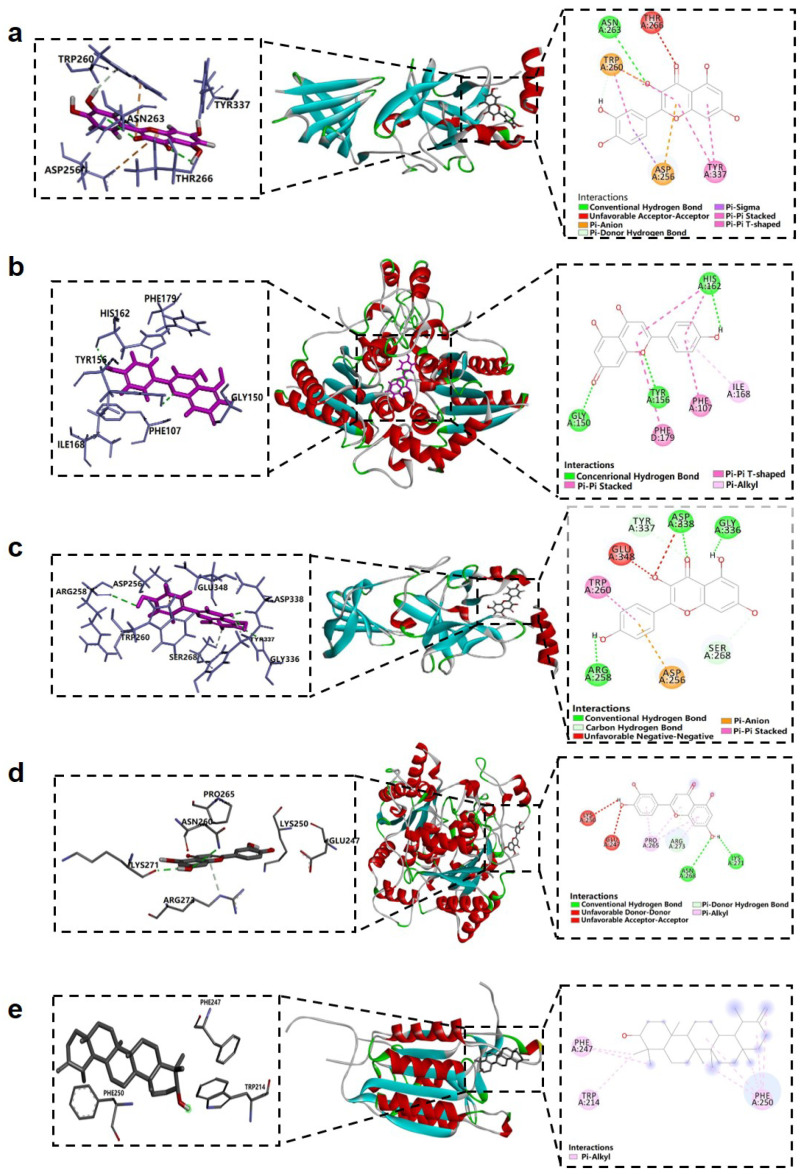
The docking map of compound–target interactions: (**a**) The docking map of quercetin–ESR1 interaction, with a binding energy of −8.9 kcal/mol. (**b**) The docking map of apigenin–EGFR interaction, with a binding energy of −7.1 kcal/mol. (**c**) The docking map of kaempferol–ESR1 interaction, with a binding energy of −9.0 kcal/mol. (**d**) The docking map of luteolin–NQO1 interaction, with a binding energy of −8.3 kcal/mol. (**e**) The docking map of taraxasterol–CASP3 interaction, with a binding energy of −6.8 kcal/mol.

Based on the results of network pharmacology prediction, six key targets—TP53, CASP3, EGFR, AKT1, ESR1, and NQO1—were selected for molecular docking with four major active compounds: quercetin, apigenin, kaempferol, and luteolin. In addition to these network-screened components, taraxasterol, a pentacyclic triterpenoid compound unique to dandelion, was also included for further analysis. Although limited target data for taraxasterol were available in public databases, its well-documented anti-inflammatory and potential antitumor activities suggest significant pharmacological relevance. Therefore, taraxasterol was incorporated into the molecular docking, molecular dynamics (MD) simulations, and in vitro validation experiments to further evaluate its anti-LC potential. Molecular docking results revealed favorable binding affinities between all selected active compounds and the core target proteins. As shown in Table 1, all docking scores were less than −6.5 kcal/mol, indicating stable interactions. It is generally accepted that the lower the binding energy, the more stable the ligand–receptor complex and the greater the binding potential. Representative docking interactions were visualized using Discovery Studio 2019 software. Quercetin bound to ESR1 (PDB ID: 3CBP [19], modeled with 243 amino acid residues) via key residues including TYR, ASN, ASP, TRP, and THR (Figure 4a). Apigenin interacted with EGFR (PDB ID: 3POZ [20], modeled with 293 amino acid residues) primarily through HIS, PHE, TYR, and GLY residues (Figure 4b). Kaempferol formed stable interactions with ESR1 (PDB ID: 3CBP, modeled with 243 amino acid residues), involving TYR, ARG, GLY, and SER (Figure 4c). Luteolin showed strong binding to NQO1 (PDB ID: 8OK0 [21], modeled with 1084 amino acid residues) through LYS, ASN, ARG, and GLU residues (Figure 4d). Finally, taraxasterol interacted with CASP3 (PDB ID: 2J32 [22], modeled with 246 amino acid residues), with PHE and TRP identified as key binding residues (Figure 4e). Collectively, these results suggest that all five active compounds possess strong binding affinities toward their respective core target proteins, supporting their potential roles in modulating LC-related signaling pathways.

### 2.3. MD Simulation Results

**Figure 5:**
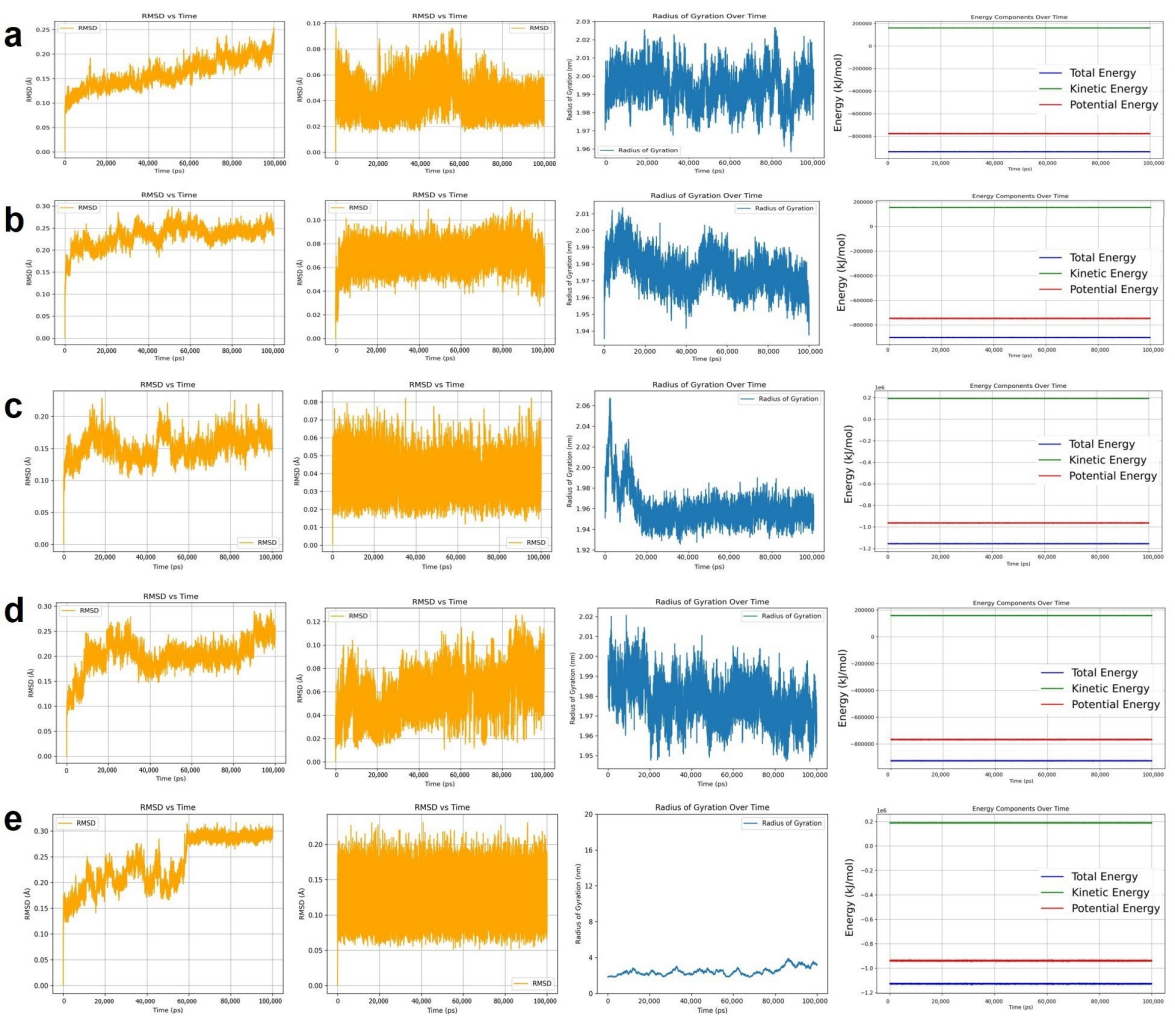
MD simulation results of five ligand–protein complexes over a 100 ns trajectory. (**a**) Quercetin–ESR1: Exhibits excellent binding stability. (**b**) Apigenin–EGFR: Exhibits excellent binding stability. (**c**) Kaempferol–ESR1: Demonstrates good binding stability. (**d**) Luteolin–NQO1: Exhibits strong interaction strength. (**e**) Taraxasterol–CASP3: Shows favorable binding stability.

To further evaluate the binding stability of the docked complexes, 100 ns MD simulations were performed for five ligand–target pairs. As shown in Figure 5a, the complex of quercetin with ESR1 exhibited excellent binding stability. The root mean square deviation (RMSD) of the protein backbone fluctuated within the range of 0.20–0.25 Å, indicating a stable protein conformation. The RMSD of the ligand remained between 0.05 and 0.10 Å throughout the simulation, suggesting that quercetin maintained a stable position within the binding site. The radius of gyration (Rg) of the complex stabilized after 20 ns, fluctuating between 2.0 and 2.2 nm, which indicates a compact and stable complex structure. Figure 5b illustrates that the apigenin–EGFR complex also demonstrated favorable binding stability. The RMSD of the protein backbone stabilized after 10 ns, ranging from 0.22 to 0.26 Å, confirming structural stability. The ligand RMSD remained within 0.06–0.08 Å after 10 ns with minimal fluctuation, indicating a consistently stable binding conformation. The Rg value stabilized after 5 ns and fluctuated slightly between 1.98 and 2.00 nm without significant trends, suggesting that the complex structure remained stable. As shown in Figure 5c, the kaempferol–ESR1 complex exhibited moderate binding stability. During the first 20 ns, the protein backbone RMSD reflected a conformational adjustment from the initial state. From 20 to 70 ns, the RMSD remained in the range of 0.15–0.18 Å, indicating dynamic equilibrium. After 70 ns, fluctuations further decreased, demonstrating improved stability. The ligand RMSD remained stable between 0.05 and 0.07 Å during 10–70 ns, indicating reliable positioning within the binding pocket. Although there was a slight increase beyond 70 ns, it remained between 0.05 and 0.08 Å. The Rg value stabilized after 10 ns and fluctuated within 1.94–1.96 nm. Figure 5d shows that the luteolin–NQO1 complex exhibited good structural stability. The protein backbone RMSD stabilized after 20 ns, with fluctuations between 0.20 and 0.30 Å. The ligand RMSD remained stable in the range of 0.06–0.12 Å after 10 ns. The Rg value of the complex also stabilized after 20 ns, fluctuating between 1.96 and 1.99 nm. As shown in Figure 5e, the taraxasterol–CASP3 complex displayed relatively stable binding characteristics. The RMSD of the protein backbone fluctuated between 0.20 and 0.25 Å from 20 to 60 ns, indicating that the system approached equilibrium. After 60 ns, the RMSD increased slightly to the range of 0.28–0.30 Å, reflecting structural adaptation followed by stabilization. The ligand RMSD remained stable between 0.15 and 0.20 Å after 10 ns. Although some fluctuation in Rg was observed, it consistently remained within the range of 2.5–3.5 nm, suggesting the overall stability of the complex. Taken together, these MD simulation results demonstrated strong and stable interactions between quercetin–ESR1, apigenin–EGFR, kaempferol–ESR1, luteolin–NQO1, and taraxasterol–CASP3. The findings provide theoretical support for the stable binding behavior of these active compounds at the molecular level and lay the groundwork for further mechanistic investigations.

### 2.4. In Vitro Evaluation of Anticancer Activity

**Figure 6:**
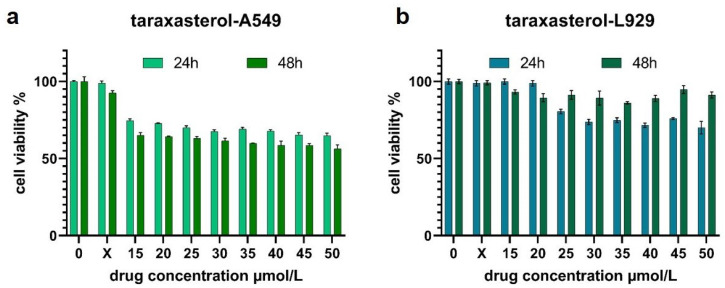
In vitro cytotoxicity evaluation of taraxasterol. (**a**) Treatment of A549 lung cancer cells with taraxasterol at 25 μmol/L for 48 h reduced cell viability to 56.26%. (**b**) Taraxasterol treatment showed no significant inhibitory effect on L929 normal cells. Data are presented as mean ± SD (N = 4).

The X-control group of taraxasterol (i.e., the solvent control group) and the pure culture medium control group exhibited no inhibitory effect on either A549 or L929 cells, indicating that the observed cell death was not induced by the solvent DMSO. As expected, the viability of A549 cells progressively decreased with increasing taraxasterol concentration and treatment duration, with the most significant inhibitory effect observed at 25 μmol/L (Figure 6a). At the same concentration, taraxasterol did not exhibit significant cytotoxicity toward normal L929 cells, with cell viability remaining at or above 80% (Figure 6b). These findings further suggest that taraxasterol may exert selective antitumor activity.

### 2.5. SEM Analysis

**Figure 7:**
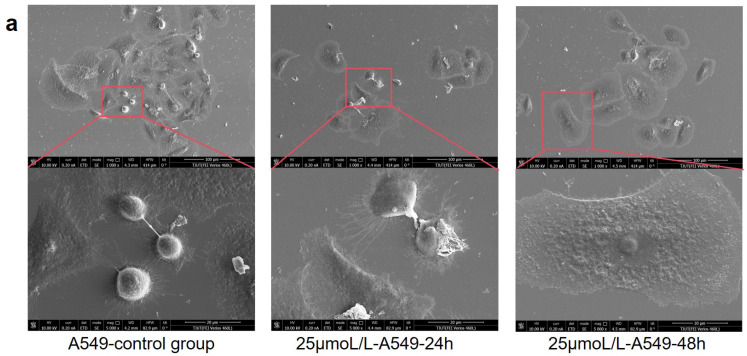

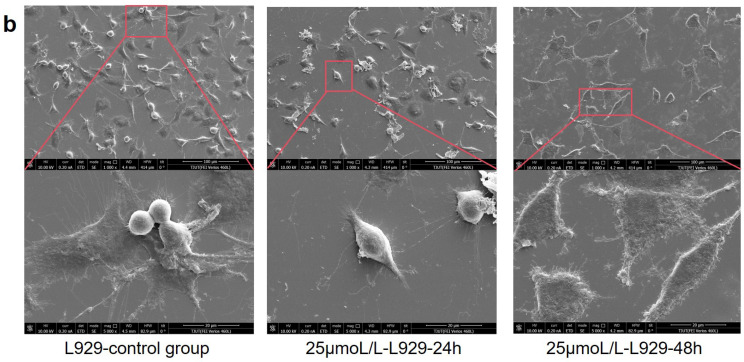
SEM analysis of the morphological effects of taraxasterol (25 μmol/L) on A549 and L929 cells. (**a**) SEM images of A549 lung cancer cells treated with taraxasterol for 24 h and 48 h, compared with the untreated control group. Significant surface damage and morphological alterations were observed over time. (**b**) SEM images of L929 cells under the same treatment conditions. No notable cytotoxic effects were observed, and overall cell morphology remained intact.

To further verify the inhibitory effect of taraxasterol on A549 cells, SEM was performed following treatment with 25 μmol/L taraxasterol on both A549 and L929 cells. As shown in Figure 7a, A549 cells exhibited significant morphological alterations after taraxasterol treatment. The degree of cell damage progressively increased with prolonged exposure. The cell surface integrity was severely disrupted, accompanied by marked cell shrinkage, increased membrane depressions, and the release of cellular debris due to partial cell rupture. These morphological features provide important visual evidence supporting the inhibitory effect of taraxasterol and offer insights into its potential mechanism of action. In contrast, as shown in Figure 7b, L929 cells treated with 25 μmol/L taraxasterol for 24 and 48 h displayed relatively intact cellular morphology. No typical features of apoptosis or necrosis were observed. Only mild surface indentations and reduced pseudopodia were noted, which may be attributed to transient cellular stress responses. These findings indicate that L929 cells exhibit good tolerance to taraxasterol at this concentration, thereby providing further morphological evidence of its selective cytotoxicity.

## 3. Discussion

In recent years, various traditional Chinese medicines (TCMs) have demonstrated promising potential in the treatment of LC. For example, *Hedyotis diffusa* [23,24] and *Scutellaria barbata* [25] are widely used in clinical practice due to their traditional functions of clearing heat and detoxifying toxins. Similarly, single herbal medicines such as *Sophora flavescens* [26], *Panax ginseng* [27], and *Tripterygium wilfordii* [28], along with their active components, have also exhibited significant antitumor effects in modern pharmacological studies. Compared to these TCMs, dandelion presents unique advantages in LC research. As a medicinal and edible herb, dandelion is considered safe and suitable for long-term use. It is rich in bioactive compounds, including flavonoids, chlorogenic acid, and ursolic acid, which can regulate key biological processes involved in LC, such as cell proliferation, apoptosis, migration, and immune response, through multi-target and multi-pathway mechanisms [29,30]. Notably, although dandelion has traditionally been used in Chinese medicine for “clearing heat and detoxifying” [31], its antitumor pharmacological mechanisms are still under investigation, highlighting its considerable research value and translational potential.

In this study, we systematically investigated the potential mechanisms of dandelion in the treatment of LC by integrating network pharmacology, molecular docking, MD simulations [32,33], and in vitro validation of taraxasterol. These approaches are well suited to elucidate the complex interactions between LC-related targets and the major bioactive compounds in dandelion. Similar strategies have recently been employed in multiple studies to explore the mechanisms of TCMs in LC therapy. Initially, we identified 49 core LC-related targets of dandelion. Network pharmacology analysis was then performed to determine the key active compounds and their corresponding target networks. Subsequently, PPI analysis, GO, and KEGG enrichment analyses were conducted to further characterize the overlapping targets. The results indicated that the anti-LC effects of dandelion involve multiple biological processes and signaling pathways, including inflammatory responses, negative regulation of cell population proliferation, responses to external stimuli, and cell cycle regulation. Based on further analysis of network topology, six hub genes—TP53, CASP3, EGFR, AKT1, ESR1, and NQO1—were identified as potential key targets underlying the anti-lung cancer effects of dandelion. Among these, ESR1 is implicated in hormone-dependent tumorigenesis [34]; EGFR is a well-characterized driver gene and therapeutic target in NSCLC [35]; NQO1 plays an essential role in maintaining redox homeostasis and modulating oxidative stress in lung tissues [36]; and CASP3, as a central executioner caspase, is crucial for the initiation of apoptosis and is involved in tumor suppression [37]. Molecular docking and MD simulations revealed strong binding affinities between several major dandelion compounds—such as quercetin, apigenin, kaempferol, and luteolin—and the core LC-related proteins. Additionally, taraxasterol, a triterpenoid compound uniquely found in dandelion, was selected for further experimental validation in vitro. Cell viability assays showed that taraxasterol had no statistically significant cytotoxic effect on A549 cells at concentrations between 25 and 50 μmol/L. To minimize potential non-specific toxicity at high concentrations, 25 μmol/L was selected as the optimal treatment concentration for subsequent SEM analysis. At this concentration, taraxasterol exhibited low toxicity to normal L929 cells (viability > 80%), indicating selective inhibitory activity against tumor cells.

Taken together, we propose that dandelion may exert anti-LC effects through synergistic regulation of multiple components and targets, ultimately modulating the expression and function of key genes. The active compounds identified in this study—particularly taraxasterol, whose effects were experimentally validated—provide a reliable theoretical and experimental foundation for further development of dandelion as a candidate agent for lung cancer therapy. However, additional in vivo experiments and pharmacokinetic studies are needed to fully evaluate its potential for clinical application.

## 4. Materials and Methods

### 4.1. Reagents and Materials

Materials: *Taraxacum mongolicum* (collected from Bozhou, Anhui Province, production batch number: 230801) and taraxasterol (purchased from Chengdu Yirei Biotechnology Co., Ltd., Chengdu, Sichuan, China, batch number: YRP003-230901) were used in this study. Cell culture dishes, 96-well plates, and 6-well plates were obtained from NEST Biotechnology. Trypsin digestion solution, fetal bovine serum (FBS), and Type I collagen were purchased from Solarbio (Beijing, China). High-glucose DMEM and phosphate-buffered saline (PBS) were obtained from Gibco (Grand Island, NY, USA). Penicillin–streptomycin solution was purchased from Servicebio (Wuhan, Hubei, China). Glutaraldehyde was provided by Fisher Scientific, and silicon wafers were supplied by Siwafer (Quzhou, Zhejiang, China). Human lung cancer cells (A549) and mouse fibroblast epithelial cells (L929) were kindly provided by Tianjin Cancer Hospital.

Instruments: An electronic analytical balance (model AR124CN, Lei Tai Instruments, Beijing, China), a high-resolution scanning electron microscope (Verios 460L, Thermo Scientific, Waltham, MA, USA), a microplate reader (Thermo Scientific, USA), and a ${\mathrm{CO}}_{2}$ incubator (Thermo Scientific, USA) were used in the experiments.

### 4.2. Experimental Procedures

#### 4.2.1. Screening of Active Compounds in Dandelion and Target Prediction

Based on the literature and modern quality control strategies for traditional Chinese medicine (TCM) [38] as outlined in the *Pharmacopoeia of China* (Part I), compounds from dandelion were screened using the Traditional Chinese Medicine Systems Pharmacology Database and Analysis Platform (TCMSP, https://www.tcmsp-e.com/, accessed on 3 July 2024) with the criteria of OB ≥ 30% and DL ≥ 0.18 [39]. Active components meeting these thresholds were identified, and their corresponding potential targets were retrieved from the TCMSP database.

Additionally, disease-related targets associated with lung cancer were obtained from the GeneCards https://www.genecards.org/ (accessed on 10 July 2024) and UniProt https://www.uniprot.org/ (accessed on 10 July 2024) databases. After intersecting the compound-related and disease-related targets, key targets of dandelion against lung cancer were identified. Cytoscape 3.8.2 software was used to construct network diagrams of “TCM–compound” and “compound–target” relationships. Network topology analysis was performed based on degree centrality, with node size reflecting degree value, to identify the most relevant active compounds.

#### 4.2.2. PPI Network Analysis

The common targets between dandelion and LC were identified using the Bioinformatics platform https://www.bioinformatics.com.cn (accessed on 6 August 2024). A PPI network of these intersecting targets was constructed using the STRING database https://string-db.org/ (accessed on 6 August 2024), with the organism set to “Homo sapiens” and a minimum required interaction score of 0.7 (high confidence). The resulting PPI network revealed the potential interactions among the target proteins for subsequent topological analysis.

#### 4.2.3. GO and KEGG Enrichment Analysis

GO and KEGG enrichment analyses were performed using the Metascape database https://metascape.org/ (accessed on 7 August 2024). The intersection targets between dandelion and lung cancer were imported into Metascape, and enriched terms were identified using a significance threshold of *p* < 0.05. GO analysis covered three categories: biological process (BP), molecular function (MF), and cellular component (CC).

#### 4.2.4. Molecular Docking

Small-molecule ligands were obtained from the PubChem database https://pubchem.ncbi.nlm.nih.gov/ (accessed on 20 August 2024), and the corresponding protein receptor structures were retrieved from the RCSB Protein Data Bank (PDB) https://www.rcsb.org/ (accessed on 20 August 2024). The molecular geometry of each ligand was optimized using Chem3D software (version 15.1). Water molecules and native ligands were removed from the protein structures using PyMOL software (version 2.3.4). Hydrogen atoms were added to both proteins and ligands using AutoDock Tools (version 1.5.6), and the binding pockets of the target proteins were identified to define the docking grid boxes. Molecular docking was then performed using AutoDock Vina (version 1.1.2) to evaluate the binding affinities between key active compounds and core target proteins. Finally, the docking results were visualized, and the specific binding sites were identified using Discovery Studio software (version 2019).

#### 4.2.5. Molecular Dynamics Simulation

To evaluate the binding stability of the protein–ligand complexes obtained from molecular docking, MD simulations were performed using the AMBER99SB force field implemented in the GROMACS software package (version 2020.6). The Single Point Charge Extended (SPC/E) three-site water model was employed to simulate the aqueous environment. Sodium (${\mathrm{Na}}^{+}$) and chloride (${\mathrm{Cl}}^{-}$) ions were added to neutralize the simulation system. After energy minimization, the system was gradually heated to 300 K. Equilibration was carried out under constant volume (NVT ensemble) and constant pressure (NPT ensemble) conditions. Subsequently, a 100 ns MD simulation was conducted under periodic boundary conditions at constant temperature and pressure. Trajectory data were recorded every 1.0 picosecond (ps). Root mean square deviation (RMSD), radius of gyration (Rg), and root mean square fluctuation (RMSF) analyses were performed to assess the conformational stability and dynamic behavior of the complexes. In addition, energy profiles were evaluated throughout the simulation to further validate system stability.

#### 4.2.6. Cell Viability Assay

Taraxasterol powder (1 mg) was precisely weighed and dissolved in dimethyl sulfoxide (DMSO) to prepare a 100 mM stock solution, which was stored at –20 °C until use. Culture medium without any treatment was used as the negative control, and a DMSO control group (X group, containing 0.005% DMSO in medium) was included. Taraxasterol was applied to cells at final concentrations of 0, 15, 20, 25, 30, 35, 40, 45, and 50 μmol/L. Exponentially growing A549 and L929 cells were diluted with DMEM to a concentration of $6\times 10^{4}$ cells/mL and seeded into 96-well plates at 100 μL per well. For each concentration, four replicate wells were set up. After incubation for 24 h, the cells were treated with the respective concentrations of taraxasterol and incubated for an additional 24 and 48 h. At the end of each treatment period, 10% MTT solution was added to each well and incubated for 4 h. Afterward, the supernatant was removed and replaced with 150 μL of DMSO to dissolve the formazan crystals. Absorbance was measured using a microplate reader, and cell viability was calculated using the following Formula (1). The cell viability curves were plotted using GraphPad Prism software (version 9.0.0).

(1) $$\mathrm{Cell}\;\mathrm{viability}=\left({\mathrm{OD}}_{\mathrm{test}}-{\mathrm{OD}}_{\mathrm{blank}}\right)/\left({\mathrm{OD}}_{\mathrm{control}}-{\mathrm{OD}}_{\mathrm{blank}}\right)\times 100\%$$

#### 4.2.7. SEM Experiment

Silicon wafers were cut into small pieces of approximately 3–5 mm in size and thoroughly rinsed with anhydrous ethanol. The wafers were then sterilized at 120 °C for 20 min. After sterilization, they were placed in culture dishes and exposed to ultraviolet (UV) light in a biosafety cabinet for 12 h to ensure complete sterility. The sterilized wafers were coated with Type I collagen solution (0.01 mg/mL) and incubated at 4 °C for 12 h. Subsequently, excess collagen was removed by rinsing twice with phosphate-buffered saline (PBS), and the wafers were air-dried before being transferred into 6-well plates for further use. A549 and L929 cells were treated with taraxasterol at a final concentration of 25 μmol/L, with untreated cells in pure medium serving as the control. Log-phase A549 and L929 cells were diluted to a density of $6\times 10^{4}$ cells/mL in DMEM and seeded onto the prepared silicon wafers in 6-well plates. After a 24-h incubation, taraxasterol was added, and the cells were further incubated for 24 and 48 h. Following treatment, the silicon wafers were gently rinsed with PBS and fixed by adding 2.5% glutaraldehyde solution directly onto the wafer surface. The samples were stored at 4 °C for 2 h for fixation, then washed with PBS to remove residual fixative. Samples were dehydrated using a graded ethanol series (30%, 50%, 70%, 90%, and 100%), with each step lasting 20 min. The 100% ethanol step was repeated three times. Finally, the samples were sputter-coated with gold to enhance conductivity and then observed under an SEM for morphological analysis.

## 5. Conclusions

This study explored the interaction between active compounds of dandelion and LC-related targets through network pharmacology analysis, molecular docking, and molecular dynamics simulations. Our research predicted the binding of key bioactive compounds from dandelion with several important LC-related targets, such as TP53, CASP3, and EGFR. Based on these analyses, we hypothesize that dandelion may exert its anticancer effects by modulating key biological processes, including tumor cell proliferation and apoptosis. The molecular docking and molecular dynamics simulation results further support the stable binding of these compounds to lung cancer targets, suggesting the potential of dandelion in lung cancer treatment.

In vitro cell assays and scanning electron microscopy (SEM) analysis confirmed that taraxasterol, a key compound of dandelion, exhibited a strong inhibitory effect on A549 lung cancer cells while showing no significant cytotoxicity to normal L929 cells. These findings provide strong experimental support for taraxasterol as a candidate compound for lung cancer therapy.

Although this study reveals the potential of dandelion in anticancer therapy, further animal experiments and clinical studies are necessary to validate its therapeutic efficacy. Additionally, future research could include comparisons with standard anticancer drugs to provide a more comprehensive evaluation of the efficacy and safety of dandelion.

In conclusion, this study provides a theoretical basis for the development of taraxasterol as a new candidate drug for lung cancer treatment and offers new insights and methods for studying the multi-target mechanisms of natural products in cancer therapy.

## Figures and Tables

**Table 1:** Binding energy data table for different compounds and targets.

Target Component	Binding Energy (kcal/mol)				
	Quercetin	Apigenin	Kaempferol	Luteolin	Taraxasterol
TP53	−8.2	−7.7	−7.6	−7.9	−7.7
CASP3	−7.0	−7.4	−7.2	−7.2	−6.8
EGFR	−6.9	−7.1	−6.7	−7.5	−7.5
AKT1	−7.7	−8.0	−8.0	−8.1	−9.7
ESR1	−8.9	−8.5	−9.0	−9.0	−8.1
NQO1	−8.8	−9.5	−8.7	−8.3	−8.1

## Data Availability

Data is contained in the paper.

## Abbreviations

The following abbreviations are used in this manuscript:

A549	Human Lung Adenocarcinoma Cell Line
ARG	Arginine
ASN	Asparagine
BP	Biological Process
CASP3	Cysteine-aspartic Protease 3
CC	Cellular Component
DL	Drug-likeness
EGFR	Epidermal Growth Factor Receptor
ESR1	Estrogen Receptor 1
GLU	Glutamic Acid
GLY	Glycine
GO	Gene Ontology
HIS	Histidine
KEGG	Kyoto Encyclopedia of Genes and Genomes
L929	Mouse Fibroblast Cell Line
LYS	Lysine
MD	Molecular Dynamics
MF	Molecular Function
MTT	3-(4,5-Dimethylthiazol-2-yl)-2,5-Diphenyltetrazolium Bromide
NPT	Isothermal Isobaric Ensemble
NQO1	NAD(P)H Quinone Dehydrogenase 1
NVT	Canonical Ensemble
OB	Oral Bioavailability
OD	Optical Density
PHE	Phenylalanine
PPI	Protein–Protein Interaction
Rg	Gyration Analysis
RMSD	Root Mean Square Deviation
SEM	Scanning Electron Microscopy
SER	Serine
SPC/E	Single Point Charge Extended
TCM	Traditional Chinese Medicine
TCMSP	Traditional Chinese Medicine Systems Pharmacology Database and Analysis Platform
TP53	Tumor Protein p53
TRP	Tryptophan
TYR	Tyrosine
UV	Ultraviolet
